# Case Report: Ofatumumab treatment in a patient with rituximab-intolerant refractory autoimmune GFAP astrocytopathy

**DOI:** 10.3389/fimmu.2023.1164181

**Published:** 2023-05-08

**Authors:** Shugang Cao, Jing Du, Sidi Pan, Juanjuan Zhang, Si Xu, Ling Wei, Yanghua Tian

**Affiliations:** ^1^ Department of Neurology, Second Affiliated Hospital of Anhui Medical University, Hefei, China; ^2^ Department of Neurology, The Second People’s Hospital of Hefei, Hefei, China; ^3^ Department of Psychology, Second Affiliated Hospital of Anhui Medical University, Hefei, China; ^4^ Department of Neurology, First Affiliated Hospital of Anhui Medical University, Hefei, China

**Keywords:** glial fibrillary acidic protein astrocytopathy, ofatumumab, rituximab, safety, efficacy

## Abstract

**Background:**

Ofatumumab, a fully humanized anti-CD20 monoclonal antibody, has shown promising efficacy in limited cases of neuromyelitis optica spectrum disorder, but there is a lack of studies on its use in autoimmune glial fibrillary acidic protein (GFAP) astrocytopathy. We present a case of refractory GFAP astrocytopathy with poor response to conventional immunosuppressants and rituximab who responded well to subcutaneous ofatumumab.

**Case report:**

The patient is a 36-year-old woman with a diagnosis of GFAP astrocytopathy and high disease activity. She experienced five relapses over three years despite immunosuppressive treatment with oral prednisone, azathioprine, mycophenolate mofetil, and intravenous rituximab. Additionally, her circulating B cells were not completely depleted during the second administration of rituximab and an allergic reaction occurred. Based on insufficient B cell depletion and allergic reaction to rituximab, subcutaneous ofatumumab was introduced. After twelve injections of ofatumumab without injection-related reactions, she had no further relapses and was sufficiently depleted of the circulating B cells.

**Discussion:**

This case illustrates the effective use and good tolerance of ofatumumab in GFAP astrocytopathy. Further studies are needed to investigate the efficacy and safety of ofatumumab in refractory GFAP astrocytopathy or those intolerant to rituximab.

## Introduction

Autoimmune glial fibrillary acidic protein (GFAP) astrocytopathy is a novel autoimmune inflammatory disease of the central nervous system (CNS) with a diagnostic biomarker of IgG against GFAP α-isoform in the cerebrospinal fluid (CSF) (1, 2). The disease is mainly characterized by involvement of the meninges, brain, spinal cord, and optic nerve, usually with a hallmark of brain linear perivascular radial gadolinium enhancement on MRI (2–4). During the remission phase, most patients respond well to steroid therapy and have a good prognosis (5, 6). However, relapses still occur in 20%−50% of patients, who require a combination of non-corticosteroid immunosuppressants to prevent future relapses (5–8). Even so, a small group of patients remain refractory, exhibiting recurrent relapses and responding poorly to conventional immunosuppressants; hence more effective biologics such as the anti-CD20 monoclonal antibody rituximab (RTX) are needed (5, 9). Ofatumumab (OFA) is a fully humanized anti-CD20 monoclonal antibody that has been approved for use in relapsing multiple sclerosis (10–12). Limited case studies have suggested similarly good efficacy of OFA in patients with neuromyelitis optica spectrum disorder (NMOSD) as salvage or first-line treatment (13–15), but there is a gap in the literature on its use in GFAP astrocytopathy. In this paper, we report a case of GFAP astrocytopathy with frequent relapses, poor response to conventional immunosuppressants, and allergic reactions to RTX. However, the patient responded well to subcutaneous OFA without complications.

## Case report

**Figure 1:**
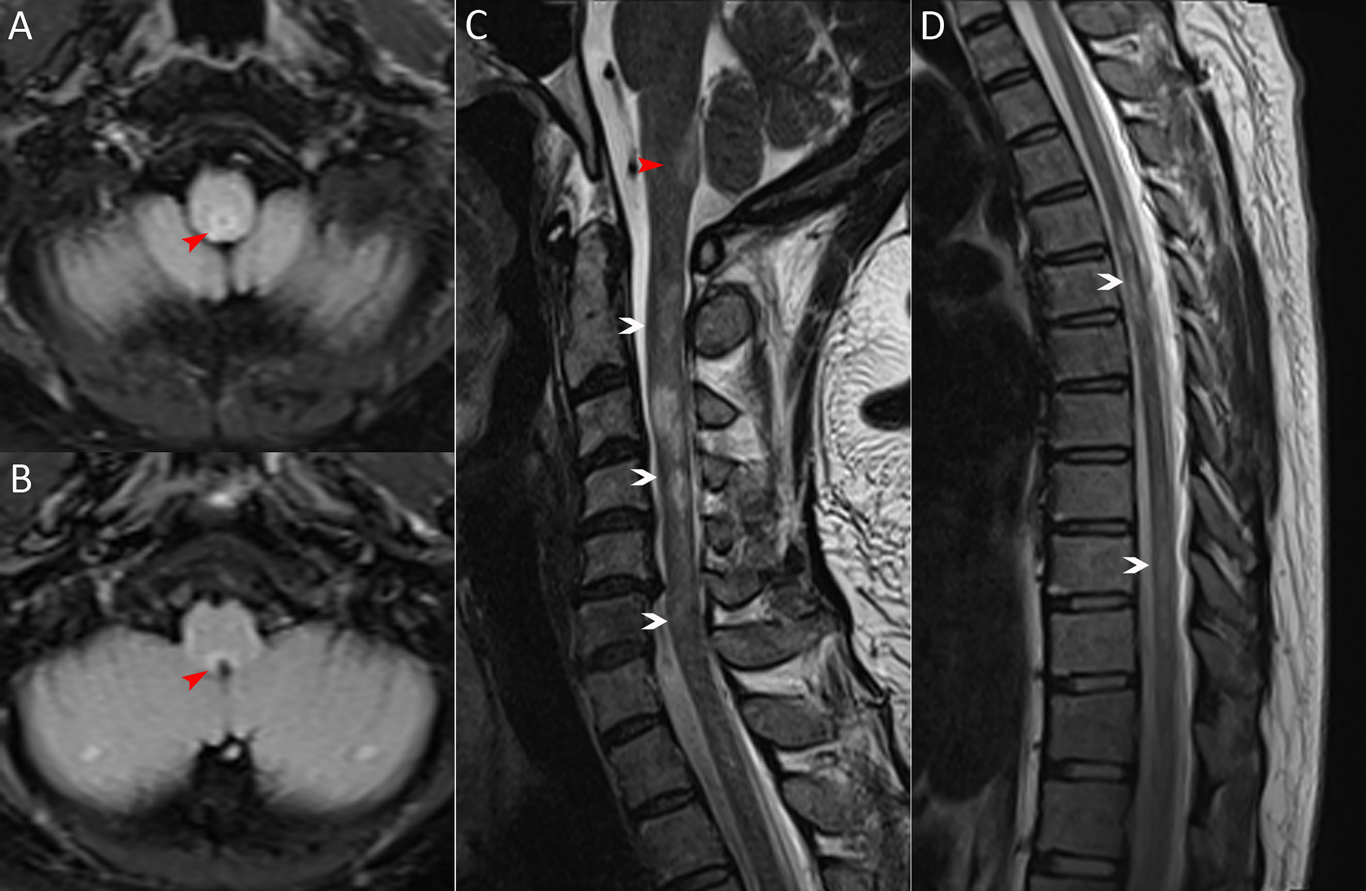
MRI scans on admission. **(A, B)** Brain MRI (axial fluid-attenuated inversion recovery image) exhibiting the area postrema lesion (red arrows, including one in Figure C). **(C, D)** Spinal cord MRI (sagittal T2-weighted image) showing demyelinating lesions in the cervical and thoracic segments (white arrows).

**Figure 2:**
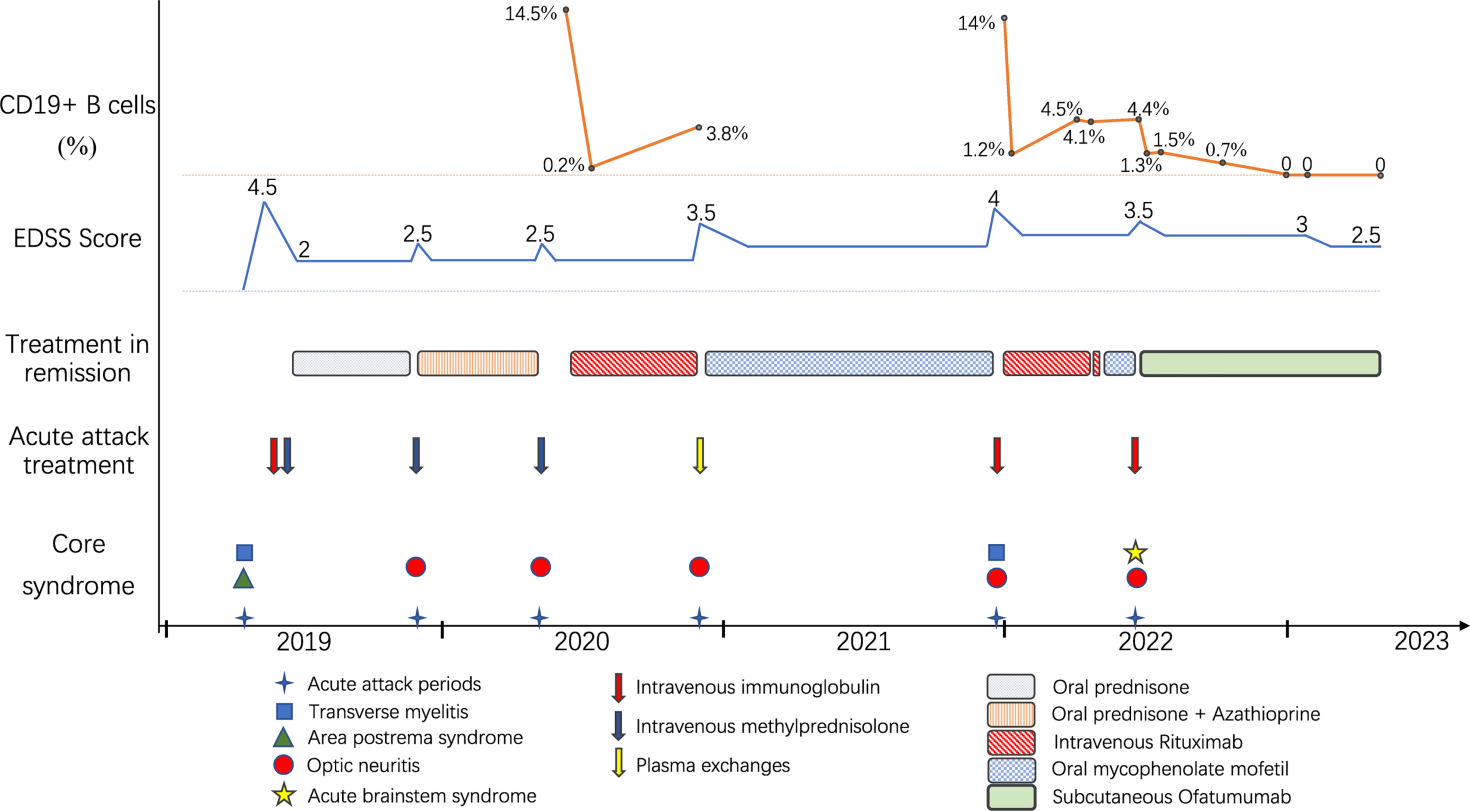
Schematic illustration of the disease course showing relapse frequency and core symptoms, disability severity, treatment regimens and CD19^+^ B-cell monitoring.

A 36-year-old woman was admitted to our hospital with chief complaints of progressive dysuria with burning pain and pruritus of extremities for two months, and spasms of left upper and lower extremities for one month. The patient reported persistent fever (maximum body temperature: 40°C) and headache with frequent hiccups and vomiting starting three months ago. Her body temperature returned to normal about one month later, but she then developed urinary retention. Neurological examination on admission revealed weakness in the left upper and lower limbs (Medical Research Council graded as 5^—^) with episodic spasms, hypoesthesia below the level of both knees, and subjective burning pain and pruritus at the extremities. The Babinski’s sign was positive bilaterally. The expanded disability status scale (EDSS) score at nadir was 4.5. The CSF test revealed a slightly elevated protein level of 666 mg/l (normal range: 150-450 mg/l), and the IgG index was 0.73 (normal range: ≤ 0.7). Oligoclonal bands were positive in CSF but negative in serum. Serum and CSF were both negative for aquaporin-4 IgG (AQP4-IgG), myelin oligodendrocyte glycoprotein IgG (MOG-IgG), and myelin basic protein IgG (MBP-IgG). Brain MRI suggested the area postrema lesion and spinal cord MRI showed demyelinating lesions in the cervical and thoracic segments (**Figure 1**). No abnormalities were detected on related tests for infection and immune indicators, anti-nuclear antibodies, anti-neutrophil cytoplasmic antibodies, anti-cardiolipin antibodies, and tumor markers. She was initially diagnosed with AQP4-IgG-seronegative NMOSD and received methylprednisolone pulse therapy (MPPT) and five infusions of intravenous immunoglobulin (IVIG). Since then, she had a marked alleviation of symptoms and was able to urinate normally. She continued to take oral prednisone to prevent relapse without any other additional immunosuppressants. Five months later, she reported right optic neuritis (ON) when she was still taking 30 mg of prednisone daily. Her visual acuity in the right eye was 0.5 with painful eye rotation. High titers of GFAP-IgG detected in CSF (1:3.2, cell-based assay) led to the diagnosis of GFAP astrocytopathy. Other central demyelinating antibodies remained negative, including AQP4-IgG, MBP-IgG, and MOG-IgG. She received oral azathioprine and prednisone for sequential therapy after undergoing MPPT again. In April 2020, she experienced a relapse of ON in her right eye (OD=0.3 and eye pain) and then received a reduced-dose RTX (500 mg) to prevent relapse. Burning pain in the extremities was dramatically relieved after RTX treatment. Unfortunately, four months later, she developed pulmonary symptoms and was diagnosed with pulmonary aspergillosis requiring antifungal treatment. Femoral head necrosis was identified during the same period of her hospitalization. A month thereafter, ON in the left eye (OS=0.5) was noted when the CD19^+^ B-cell percentage was 3.8% of lymphocytes. She received plasma exchanges and then switched to mycophenolate mofetil (MMF) (1.0 g orally, twice daily). She maintained stable conditions for one year and then developed severe bilateral vision loss (OD: light perception and OS=0.3) with eye pain and increased numbness in the extremities (EDSS=4). Visual evoked potentials suggested no P100 waveform elicited in both eyes. Optic nerve MRI suggested bilateral optic nerve dilatation with gadolinium enhancement, and spinal cord MRI suggested C3-4 level enhanced lesions. She was treated with IVIG and received another reduced-dose RTX regimen (100 mg intravenously on Day 1 and then 500 mg intravenously on Day 2). Although there was an improvement in vision, she developed an anaphylactic-like reaction 7 days after the initial dosage and was hospitalized in the neurological intensive care unit due to anaphylaxis. After 2.5 months, the CD19^+^ B-cell percentage rose to 4.5% and was maintained at 4.1% for the retest of B-cells 5 days later. She strongly requested another RTX infusion due to fear of relapse. Unfortunately, she had an allergic reaction (laryngeal edema and dyspnea) a few minutes after RTX administration, even though we had given her premedication including antihistamines, antipyretics and corticosteroids before administration in consideration of infusion reactions. She switched back to MMF for relapse prevention. However, two months thereafter, she again experienced vision loss in her right eye (finger count/20 cm) and limited adduction of the right eye with diplopia. Brainstem lesions were suspected but not identified on brain MRI. Optic nerve MRI suggested right optic nerve enhancement, suggesting a relapse. After treatment with IVIG, subcutaneous OFA (20 mg on Days 1, 7, and 14, and 20 mg monthly thereafter) was introduced on 28 June 2022 due to persistent relapses and adverse events associated with RTX. One month after receiving OFA administration, her visual acuity returned to 0.2, numbness with burning pain and pruritus in the extremities was significantly relieved, and spasms in the left upper and lower extremities disappeared. The CD19^+^ B-cell count decreased significantly. As of 19 April 2023, she had received twelve subcutaneous injections of OFA with no relapse, a CD19^+^ B-cell percentage of 0, and a serum IgM level of 17.0 mg/dL (**Figure 2**).

## Discussion

A small group of GFAP astrocytopathy patients are insensitive to both glucocorticoids and conventional immunosuppressive agents and still have recurrent relapses, requiring highly effective biological agents such as anti-CD20 monoclonal antibodies (5, 9). RTX is the most commonly used human-mouse chimeric anti-CD20 monoclonal antibody and can be used off-label for GFAP astrocytopathy (5, 9). Although RTX has shown great efficacy in preventing relapses of autoimmune and inflammatory CNS disease, its use can be limited by serious infusion-related reactions, anti-drug antibodies, and infection risk (16, 17). In the present case, the patient developed pulmonary infection and allergic reactions after RTX administration. Moreover, diminishing depletion of B cells after the second dose (from 14% to 1.2% after 600 mg RTX) was noted in comparison to the first dose (from 14.5% to 0.2% after 500 mg RTX), and B cells proliferated excessively in a short period of time. Lack of efficacy has been suggested to be related to the presence of anti-drug antibodies that prevents the binding of RTX to CD20 (13, 17), so it was speculated that the patient might have developed anti-drug antibodies, causing insufficient depletion of B cells and adverse events such as hypersensitivity infusion reactions.

OFA is a fully humanized anti-CD20 monoclonal antibody that can be self-administrated by patients. Unlike the epitopes recognized by RTX, OFA acts by binding to two unique epitopes on CD20-expressing B cells to induce pathogenic B cell depletion *via* complement-mediated CD20^+^ B cell lysis and antibody-dependent cell-mediated cytotoxicity (12). The fully humanized design prevents the risk of anti-drug antibody production, which greatly prevents treatment failure and has fewer injection-related systemic reactions than infusion-related ones (12). As reported in our case, the patient responded poorly and had an allergic reaction to RTX. Fortunately, she responded well to OFA, had no injection-related adverse reactions, and was sufficiently depleted of circulating CD19^+^ B cells. Even so, the patient has not been on OFA for long enough to observe the long-term efficacy and safety of OFA in GFAP astrocytopathy.

In conclusion, the first use of subcutaneous OFA in a patient with refractory GFAP astrocytopathy here suggested that OFA might be a well-tolerated and effective alternative. Further studies are needed to investigate the safety and efficacy of OFA in refractory GFAP astrocytopathy or those intolerant to RTX.

## Data availability statement

The original contributions presented in the study are included in the article/supplementary material. Further inquiries can be directed to the corresponding author.

## Ethics statement

The studies involving human participants were reviewed and approved by the Institutional Review Board of the Second Affiliated Hospital of Anhui Medical University. The patients/participants provided their written informed consent to participate in this study. Written informed consent was obtained from the individual(s) for the publication of any potentially identifiable images or data included in this article.

## Author contributions

SC drafted the manuscript. SC, JD, SP, JZ and SX prepared the materials, collected, and analyzed the data. LW and YT revised the manuscript. All authors contributed to the article and approved the submitted version.
